# Analysis of the Correlation Between *Toxoplasma gondii* Seropositivity and Alzheimer’s Disease

**DOI:** 10.3390/pathogens13111021

**Published:** 2024-11-20

**Authors:** Jianjun Wang, Ping Lin, Dan Li, Biyu Yang, Jiaqi Wang, Meng Feng, Xunjia Cheng

**Affiliations:** 1 Shanghai Institute of Infectious Disease and Biosecurity, Fudan University, Shanghai 200032, China; 22211020024@m.fudan.edu.cn; 2 Department of Medical Microbiology and Parasitology, School of Basic Medical Sciences, Fudan University, Shanghai 200032, China; linpingsun2000@aliyun.com (P.L.); 18301010019@fudan.edu.cn (B.Y.); 18301010020@fudan.edu.cn (J.W.); 3 Department of Clinical Laboratory, Shanghai Mental Health Center, Shanghai Jiao Tong University School of Medicine, Shanghai 200030, China; sichu.youdang@163.com

**Keywords:** Alzheimer’s disease, *Toxoplasma gondii*, cyst wall protein 1, multivariate logistic regression model

## Abstract

Alzheimer’s disease (AD) is a multifactorial brain disorder and infectious diseases are considered as one of the predisposing factors for AD. *Toxoplasma gondii*, an obligate intracellular parasitic protozoan, is suspected of being associated with AD. Serum samples were collected from 109 AD patients and 114 age-matched healthy controls. ELISA was performed using recombinant *T. gondii* cyst wall protein 1 (CST1) to detect *T. gondii* antibodies. A parallel experiment was performed with Toxoplasma gondii tachyzoites lysate protein. To analyze whether factors associated with the onset of AD included chronic *T. gondii* infection, a multivariate logistic regression model was applied, further validating the correlation between chronic *T. gondii* infection and AD. AD patients exhibited significantly higher levels of Toxoplasma-specific antibodies in their serum compared to the control group, with statistically significant differences (*p* < 0.05). Multivariate logistic regression analysis revealed that *Toxoplasma* infection is a risk factor for AD (*p* < 0.01), and the CST1 antigen can significantly improve the model’s performance in predicting the occurrence of AD. The results indicate that chronic infection with *Toxoplasma gondii* could be one of the risk factors for the development of AD, potentially predisposing individuals with underlying health conditions to the disease. This further validates the correlation between *Toxoplasma gondii* and AD.

## 1. Introduction

As the economy and society continue to develop, living standards and healthcare conditions have improved, leading to an increased life expectancy. However, this has also intensified the issue of population aging. With aging comes a significant medical and social challenge in the form of age-related diseases, particularly Alzheimer’s disease and other types of dementia.

Alzheimer’s disease is a neurodegenerative disease with insidious onset and progressive development. Its main pathological features include the abnormal deposition of beta-amyloid protein in the brain, leading to plaque formation, and the abnormal phosphorylation of tau protein, resulting in neurofibrillary tangles [1,2]. The exact cause of Alzheimer’s disease remains unclear. *T. gondii*, an obligate intracellular parasitic protozoan, is widely distributed and capable of infecting various mammals, posing an opportunistic infection risk to humans. Due to its neurotropic nature, chronic *T. gondii* infection can subtly damage brain tissue [3,4], leading to subtle brain infections with no significant clinical symptoms, and may result in behavioral disorders such as agitation, impulsivity, and risky behavior [5].

In recent years, growing research suggests that *T. gondii* infection may be associated with various neurological and psychiatric disorders, including Alzheimer’s disease [6,7,8]. For instance, anxiety exacerbation is a typical symptom associated with *T. gondii* seropositivity [9], which is also connected to a range of neurological and psychiatric behavioral changes, including Alzheimer’s disease, autism, epilepsy, and schizophrenia [10,11].

It is estimated that approximately one-third of the global population has been seropositive for *T. gondii*, but the prevalence varies significantly across countries and regions, ranging from 10% to 80% [12]. In Central and Southern Europe, seropositivity rates can reach intermediate levels (30–50%), while they are higher in Latin America and tropical African countries [13]. In China, epidemiological studies have shown considerable variation in *T. gondii* seropositivity between different species and provinces. In Fujian, the seropositivity rate from serological tests ranges from 4% to 8%, with the nationwide distribution estimated to be 7.2–23.41% [14,15]. Given the global distribution and extensive natural reservoirs of *T. gondii*, along with its neurotropic nature, investigating whether *T. gondii* is a risk factor in the progression of Alzheimer’s disease is crucial for both preventing Alzheimer’s disease and deepening our understanding of this often-underestimated parasite.

During chronic latent infection of *T. gondii*, the parasite exists as bradyzoites within tissue cysts, which are modified parasitophorous vacuoles. The encapsulation of bradyzoites by the cyst wall is crucial for immune evasion, survival, and dissemination [16]. Notably, tissue cysts formed during the bradyzoite stage in host tissues are a hallmark of chronic infection [17]. The composition of the *T. gondii* cyst wall is complex, primarily consisting of CST1 and dense granule proteins (GRAs) [18]. CST1, a large glycoprotein with a mucin-like structural domain, is present at multiple stages of the *T. gondii* life cycle and is essential for constructing a complete cyst wall and providing structural rigidity [16,19]. From a biological perspective, CST1, which contains a mucin-like domain and is O-GalNAc-glycosylated, resembles the cyst wall protein SRS13 from the SRS protein family. CST1 may mask other antigenic epitopes on the parasite surface, thereby reducing recognition by the host immune system. The cyst wall could rapidly uptake nutrients, and the mucin-like domain might be involved in nutrient binding or transport, supporting the survival and potential replication of bradyzoites within the cyst [20]. These mucin-like domains likely protect bradyzoites within the cyst from external environmental influences, making CST1 crucial for the survival of *T. gondii*.

Studies using ELISA, Western blot, and indirect immunofluorescence analysis have confirmed that CST1 possesses significant immunogenicity and is recognized by the host immune system [21]. Strong humoral immune responses against CST1 are present in the sera of animals and humans chronically infected with *T. gondii*, indicating that CST1 induces host-specific antibodies. Importantly, CST1 expression during the cyst stage is specific, reducing cross-reactivity with other antigens and enhancing the specificity of CST1 as a marker for chronic *T. gondii* infection. Therefore, CST1 was selected as a biomarker for identifying chronic *T. gondii* infection due to its critical role in the cyst wall, its ability to induce strong humoral immune responses, its specificity and immunogenicity, and its potential application in serological testing. Given the importance of the CST1 protein in the serodiagnosis of chronic *T. gondii* infection and in evaluating *T. gondii* seropositivity, the present study examined *T. gondii* antibodies using ELISA with recombinant CST1. Serum samples from 109 Alzheimer’s disease patients and 114 healthy controls were collected and examined.

## 2. Materials and Methods

### 2.1. Study Design and Ethical Considerations

**Figure 1:**
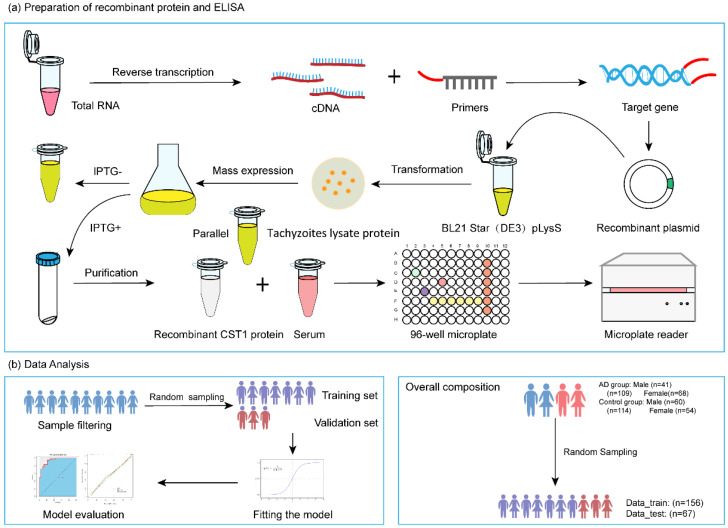
Overall study design and sampling strategy. Schematic diagram of workflow for recombinant protein preparation (**a**) and data analysis (**b**).

The study was conducted in Shanghai in 2022. The main work of this study includes serum collection from patients and healthy controls, preparation of the recombinant protein, an enzyme-linked immunosorbent assay, and data analysis (Figure 1).

Inclusion criteria for the AD group included the presence of Alzheimer’s disease (both early and late onset) and written informed consent from the patient or legal representative. Patients with other types of psychiatric disorder, such as schizophrenia, were excluded. Exclusions also included patients with other types of dementia, such as vascular dementia, patients with major systemic diseases, and patients with a history of drug or alcohol abuse. The healthy control group was age-restricted, selecting non-AD individuals aged 55 years or older as controls. The final sample size included a total of 223 individuals, with 109 AD patients and 114 healthy controls. Considering sample representativeness, the serum samples were provided by the Shanghai Mental Health Center, affiliated with Shanghai Jiao Tong University. These samples were collected and preserved during health examinations of inpatients at the center, with consent obtained from the patients and their families. Participants were selected by simple random sampling from those who met the inclusion criteria. The study received approval from the Ethics Committee of the Shanghai Mental Health Center, Shanghai, China (ethics review number 2022ky-76) to use the serum samples, and written informed consent was obtained from the patients or their families.

### 2.2. Materials

The serum samples were provided by the Shanghai Mental Health Center, affiliated with Shanghai Jiao Tong University. The total number of participants providing serum samples was 223, including 109 AD patients and 114 healthy controls. The AD group had an average age of 76.9 years, with 68 females, making up 62.4% of the group. The healthy control group had an average age of 70.7 years, with 54 females, representing 47.4% of the group (Table 1). The tachyzoites lysate protein of the *T. gondii* strain RH used in the parallel experiments were produced by Virion\Serion, Germany (BA110VS). The recombinant CST1 protein was prepared through the efficient expression of target genes in BL21 Star (DE3) pLysS competent cells. After washing the inclusion bodies ten times with IB Wash Buffer, 30 mg of the inclusion body protein was redissolved in IB Solubilization Buffer at a concentration of 5–10 mg/mL. The solution was then centrifuged at 10,000× *g* for 10 min, and the supernatant was dialyzed and subjected to refolding dialysis. Following sterile filtration and affinity chromatography, pure recombinant CST1 protein was obtained for detecting antibodies against *T. gondii*. ELISA verification using serum from patients with *Toxoplasma* encephalitis and healthy human serum demonstrated that CST1 could be recognized by serum from *Toxoplasma* encephalitis patients but not by healthy human serum.

### 2.3. Methods

Based on the *T. gondii* RH strain gene sequence published in Genbank, primers were designed with appropriate restriction enzyme sites added to the 5′ ends of both upstream and downstream primers. PCR amplification was performed using these primers to obtain the gene fragment encoding the CST1 protein. The primers used were Tg-CST1-S (Nde): 5′-CCCCATATGATGAAGAAAATAGAG-3′ and Tg-CST1-AS (Xho): 5′-CCCCTCGAGTTAGTAGACTCTGGTGAC 3′. The DNA fragment encoding CST1 was then ligated into the expression vector pET-19b. The plasmid with correct insert orientation was transformed into *Escherichia coli* strain BL21 Star (DE3) pLysS and induced with 1 mmol of isopropylthiogalactoside (IPTG) for protein expression in a Luria-Bertani medium. Following bulk expression, the recombinant CST1 protein was harvested as an inclusion body protein and purified for antibody detection against *T. gondii*.

ELISA was performed using serum samples in 96-well flat-bottomed microtiter plates (Greiner Bio One, Frickenhausen, Germany), with each sample tested in triplicate. The ELISA plate was coated with 100 μL of coating buffer containing 0.1 μg of purified recombinant CST1 protein or 0.5 μg tachyzoites lysate protein and incubated overnight at 4 °C. Each well was washed with PBS-Tween and blocked with 3% skim milk. A 100 μL aliquot of serum, diluted 1:400 in PBS (from Alzheimer’s patients and healthy controls), was added, and the mixture was incubated at room temperature for one hour. After washing, 100 μL of HRP-labeled anti-human IgG antibody (1:1000 dilution) was added and the mixture incubated at room temperature for one hour. Following another wash, 200 μL of an o-Phenylenediamine-based development solution was added, and the reaction was halted with H_2_SO_4_ after 30 min. Absorbance was measured at 490 nm using an ELX800 universal microplate reader (Bio-Tek Instruments, Inc., Winooski, VT, USA). A positive result was defined as an absorbance value of 490 nm greater than the mean plus two standard deviations (SD) of the healthy control group.

### 2.4. Statistical Analysis

Statistical analysis was conducted using GraphPad Prism 9, SAS software version 9.4, and R version 4.4.1. A total of 109 Alzheimer’s disease patients from the Shanghai Mental Health Center were selected and matched with a control group (114 cases). Demographic data and reactivity levels of serum *T. gondii*-related antigens were collected from both the Alzheimer’s group and the control group. Multivariate logistic regression and a nomogram model were used to analyze whether factors associated with the onset of Alzheimer’s disease included chronic *T. gondii* infection. Receiver Operating Characteristic curves (ROC), the Hosmer–Lemeshow test, and calibration curves were used to evaluate the classification performance, goodness of fit, and generalization ability of the model, further validating the correlation between chronic *T. gondii* infection and Alzheimer’s disease.

The Mann–Whitney U test and the chi-square test were initially used to assess differences in age and gender between the case and control groups. After data cleaning and processing, the dataset was split into a training and validation set in a 7:3 ratio, with the training set used for modeling and the validation set for model validation. Risk factors for Alzheimer’s disease occurrence were analyzed using a logistic regression model, and ROC curves were generated. The probability (Pr) and relative risk were calculated using the following equations:

$$\mathrm{Pr}\;(Y=1|X_{1},\;X_{2},\;\ldots ,\;X_{m})=\frac{exp(\beta_{0}+\beta_{1}x_{1}+\beta_{2}x_{2}+\ldots +\beta_{m}x_{m})}{1+exp(\beta_{0}+\beta_{1}x_{1}+\beta_{2}x_{2}+\ldots +\beta_{m}x_{m})},\;\mathrm{RR}=P_{1}/P_{0}$$

where Y = 1 if the participant tested positive for Alzheimer’s disease, and Y = 0 if the participant tested negative. P_0_ represents the probability of a positive result in the nonexposed condition, while P_1_ is the probability of a positive result in the exposed condition. The nomogram and calibration curve for the predictive model were plotted using the R package (rms), and the Hosmer–Lemeshow goodness-of-fit test was performed to assess the model’s accuracy.

## 3. Results

### 3.1. Demographics

**Figure 2:**
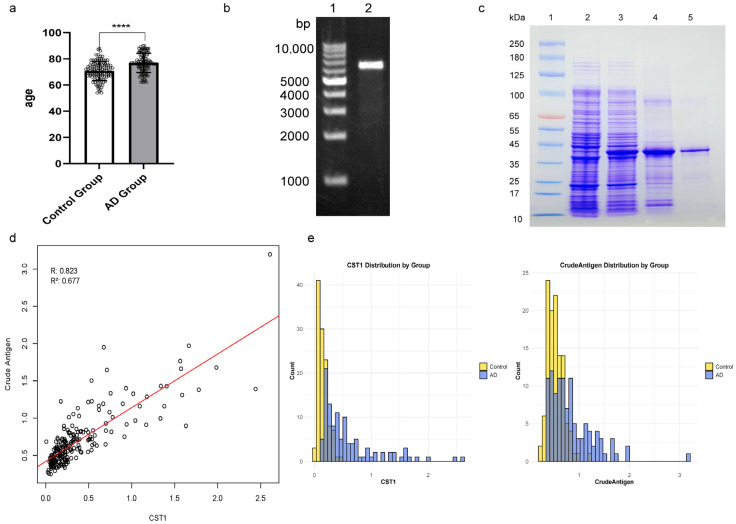
Data presentation and purification of the *Toxoplasma gondii* CST1 antigen. (**a**) Age distribution of the Alzheimer’s and control groups. Statistical significance is indicated by **** *p* < 0.0001, as determined by an unpaired *t*-test with Welch’s correction. (**b**) Electrophoresis of the recombinant plasmid. Lane 1 shows the 1kb DNA Ladder, and Lane 2 shows the recombinant plasmid. (**c**) SDS-PAGE of the prokaryotically expressed and purified recombinant CST1. Lane 1: the BiostepTM Prestained Protein Marker (catalog: 180-6006), lane 2: noninduced bacterial fluid, lane 3: induced bacterial fluid, lane 4: the inclusion body of CST1, lane 5: the refolded CST1 protein after affinity chromatography. (**d**) Linear regression between CST1 and tachyzoites lysate protein, showing an R value of 0.823 and R^2^ of 0.677. (**e**) Categorical histograms of CST1 and tachyzoites lysate protein distribution across groups.

Of the 223 participants, 109 were Alzheimer’s disease patients, who were generally older than the participants in control group. To ensure a degree of age matching, the control group was selected with an age cutoff of 55 years and above. Despite this, the overall age distribution of the control group remained lower than that of the Alzheimer’s disease (AD) group (Table 1, Figure 2a). Consequently, age must still be considered as a variable in the data analysis model. Table 1 shows the demographic characteristics of the participants in this study. The mean age of the AD group was 76.9 years (SD = 7.3 years), significantly higher than the mean age of 70.7 years (SD = 7.2 years) in the control group (*p* < 0.001). The proportion of females in the AD group was 62.4%, compared to 47.4% in the control group, with a significant difference between the groups.

### 3.2. Expression of Recombinant CST1 and Parallel Experiment with Tachyzoites Lysate Protein

The CST1 gene fragment, approximately 1122 bp in length, was amplified from *T. gondii* cDNA and successfully linked into the pET-19b expression vector (Figure 2b). Sequencing of the recombinant plasmid revealed a 99% homology with the CST1 gene of the *T. gondii* RH strain. The target plasmids were transformed into BL21 Star (DE3) pLysS cells, and inclusion bodies were harvested following inducement. Protein refolding was performed, and the refolded proteins were further purified using Ni-NTA affinity chromatography. The recombinant CST1 was then analyzed by sodium dodecyl sulfate-polyacrylamide gel electrophoresis under reducing conditions (Figure 2c). An enzyme-linked immunosorbent assay of *T. gondii* tachyzoites lysate protein with IgG in serum was used by us as a parallel experiment for the recombinant glycoprotein CST1, which showed a strong positive linear correlation (Figure 2d). Based on the scatter plot and fitted line, the R value (correlation coefficient) of 0.823 indicates a high positive correlation between CST1 and the tachyzoites lysate protein level, suggesting that CST1 could potentially serve as a marker for *T. gondii* infection. The R^2^ value of 0.677 implies that CST1 levels explain approximately 67.7% of the variability in tachyzoites lysate protein levels. This is a moderate to high degree of fit in biological data.

### 3.3. Reactivity Assay of CST1 and Tachyzoites Lysate Protein with Serum

Reactivity of the recombinant CST1 with serum samples was assessed using ELISA. The mean ELISA value for the AD group was 0.590 (SEM, 0.048), which was statistically higher than the mean value of 0.147 (SEM, 0.008) for the control group. From the categorical histograms, we can draw a few key conclusions (Figure 2e). First, the distribution of CST1 shows a notable difference between the AD and control groups. CST1 values in the control group are concentrated in a lower range, while in the AD group, higher values are more prevalent, suggesting an elevated level of CST1 in AD patients that may be associated with AD incidence. For the tachyzoites lysate protein, while there is an overlap in distribution between the AD and control groups, the AD group exhibits a higher frequency in the upper ranges, whereas the control group is more evenly distributed or skewed towards lower values. Additionally, the shapes of these distributions provide further insight: in both groups, CST1 shows a relatively single-peaked distribution with distinct peaks, suggesting its potential as a differentiating marker for AD. Tachyzoites lysate protein, on the other hand, is more dispersed in the control group and possibly multimodal, indicating that it may not distinguish AD from controls as clearly as CST1. In summary, these histograms indicate that CST1 may have strong discriminative power in identifying AD patients.

### 3.4. Contribution of CST1 Variables to the Multivariate Logistic Regression Model

Table 2 compares the performance of two models, one with CST1 and one without, across several evaluation metrics. The sensitivity of the model with CST1 (0.9032) is significantly higher than that of the model without CST1 (0.7097), and the specificity of the model with CST1 (0.8286) is higher than that of the model without CST1 (0.6571), indicating that the model with CST1 is better at correctly identifying AD patients and healthy controls. The false positive rate for the model with CST1 (0.1714) is lower than that of the model without CST1 (0.3429), and the false negative rate for the model with CST1 (0.0968) is also lower than the model without CST1 (0.2903). The accuracy of the model with CST1 (0.8636) is much higher than that of the model without CST1 (0.6818), reflecting the overall better performance of the model with CST1. Balanced accuracy is also higher in the model with CST1 (0.8659 vs. 0.6834), showing that it performs more equally well across both classes. The Kappa statistic for the model with CST1 (0.7278) is higher than for the model without CST1 (0.3648), indicating better agreement with the actual labels. The positive predictive value (PPV) and negative predictive value (NPV) are both higher in the model with CST1 (0.8235 and 0.9062, respectively) compared to the model without CST1 (0.6471 and 0.7188), meaning the model with CST1 is more reliable when predicting both AD patients and healthy controls. Overall, the model with CST1 outperforms the model without CST1 across all metrics, demonstrating that adding CST1 improves the model’s predictive power.

### 3.5. Assessment of Interaction Terms for the Final Model Selection

As shown in Table 3, Model 1 has no interaction term and contains only CST1, age, and gender, and the OR for CST1 is 4.75 and highly significant, showing its significant positive influence on the risk of AD. In Model 2, the “sex (female)*CST1” interaction term was added, but this interaction term was not significant (*p* = 0.874), with an OR close to 1, suggesting a weak influence of gender on the effect of CST1. Model 3 was built on Model 2 by adding the “age*CST1” interaction term, which shows an OR slightly greater than 1, but it is not significant (*p* = 0.110), indicating that age has a minimal effect on CST1. Model 4 contained a triple interaction term, “age*sex (female)*CST1”, which was not significant, with an OR close to 1, indicating that the interaction term did not provide significant enhancement to the model. Although the inclusion of interaction terms in multivariate logistic regression models is often helpful for exploring relationships between variables—for instance, assessing whether the effect of CST1 on Alzheimer’s disease risk varies by sex or age—our analysis showed that all interaction terms had high *p*-values (greater than 0.05), indicating no significant interactions. Model 1, which includes only the main effects, appeared to be the most concise and effective model for understanding the relationship between CST1 and Alzheimer’s disease risk. Therefore, the final model we selected was Model 1. The final model indicated that the CST1 antibody level (OR = 4.75), reflecting chronic *T. gondii* infection, and age (OR = 1.10) are risk factors for AD (*p* < 0.01).

### 3.6. Predictive Value of the Serum Antibody Level of the CST1 Antigen for the Occurrence of AD

The Hosmer–Lemeshow goodness-of-fit test was performed to assess the multivariate logistic regression model and the final *p*-value obtained was 0.4528, which is greater than 0.05. Therefore, it can be concluded that there is no significant difference between the predicted and observed probabilities, indicating that the model fits the data well.

**Figure 3:**
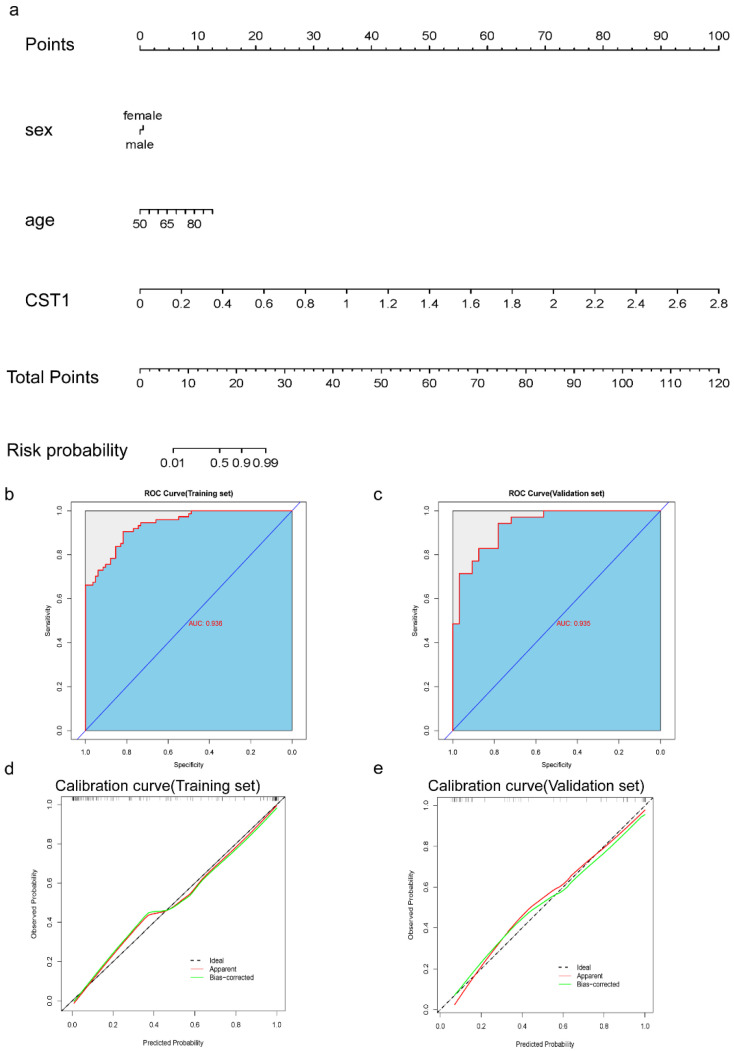
Visualization of the logistic regression model prediction results and evaluation of the model’s performance. (**a**) Nomogram for the prediction of risk of Alzheimer’s occurrence based on the logistic regression model. The scores of all variables were summed to obtain a total score corresponding to the risk prediction axis, which is the probability of the occurrence of Alzheimer’s disease. (**b**,**c**) The ROC curves for the training and validation sets; the areas under the ROC curve (AUC) are 0.936 and 0.935, respectively. (**d**,**e**) The calibration curves for the training and validation sets. The ideal line indicates the ideal situation, where the model predictions are in perfect agreement with reality. The apparent line represents the model’s performance on the original training dataset, which has not been subjected to any external validation or correction and shows the model’s prediction accuracy on the training set. Bias-corrected is the model performance after we corrected the overfitting phenomenon using the 1000-times resampling method (bootstrap).

The nomogram indicated that the CST1 antibody level, along with age, can serve as predictive indicators for the onset of AD (Figure 3a). To evaluate the performance of the multivariate logistic regression model, the dataset was divided into training and validation subsets with a 7:3 split ratio using a random sample. The training set demonstrated an ROC value (AUC) above 0.936 (Figure 3b), while the validation set exhibited an ROC value (AUC) above 0.935 (Figure 3c). After achieving good model discrimination, the predictive ability of the model was further validated using 1000 bootstrap samples from the training set and the validation set respectively, and the calibration curves were plotted. The calibration curves of the training set closely aligned with the ideal line, with only six deviations observed, indicating that the model fits quite well on the training set and is able to estimate the predicted probabilities well (Figure 3d). From the calibration curve of the validation set, we can see that both the “Apparent” (actual) and “Bias-corrected” curves are very close to the ideal line (Figure 3e). This indicates that the model’s calibration is good on the validation set, meaning that the predicted probabilities closely match the observed probabilities without significant overestimation or underestimation. Such calibration curves that closely follow the ideal line generally suggest that the model’s predictions are accurate and consistent across different probability ranges, demonstrating strong generalizability across different data sets. The results demonstrate that the model has a predictive ability to some degree, and that the CST1 antibody level can improve the predictive ability of the model.

## 4. Discussion

AD is the most prevalent form of dementia, accounting for 60–70% of all dementia cases. As reported by the World Health Organization, over 55 million people worldwide suffered from dementia as of 2019, with nearly 10 million new cases emerging every year. AD and other forms of dementia rank as the seventh leading cause of death, significantly contributing to disability and dependence among the elderly globally. In China, there are approximately 13,243,950 cases of AD and other forms of dementia, with an incidence rate of 56.47–207.08 per 100,000, a prevalence rate of 924.1 per 100,000, and a mortality rate of 22.5 per 100,000 [22,23,24]. Despite extensive research, the exact pathogenesis of AD remains unclear, and current theoretical models have not fully elucidated its mechanisms [25]. This has led to significant challenges in developing effective prevention and treatment strategies. Exploring risk factors is crucial, not only for preventing AD but also for complementing and improving the understanding of its pathogenesis. Recognized risk factors for AD include age [26], genetics [27], cardiovascular disease [28], traumatic brain injury [29], and environmental factors [30]. Recent research has increasingly pointed to various pathogens, including *T. gondii*, as playing a significant role in the onset and progression of AD. In particular, the infectious etiology of late-onset AD (LOAD) has been proposed over the past three decades, with several studies suggesting that certain pathogens are major contributors to AD and exploring antimicrobial therapy as a potential treatment option [31,32].

Serological testing is a widely accepted method for diagnosing *Toxoplasma* infection [33]. By comparing the seropositivity rates of *Toxoplasma* antibodies between the case and control groups, considering the associated pathological mechanisms, one can infer the epidemiological association between *Toxoplasma* and cognitive neuropsychiatric disorders. For instance, patients with chronic *Toxoplasma* infection have been found to have a significantly higher rate of traffic accidents compared to a control group [34]. Asymptomatic chronic *Toxoplasma* infection may represent a serious and often-underestimated public health issue. In chronic infections caused by *T. gondii*, the bradyzoite stage does not lead to host cell rupture quickly but forms thick-walled cysts containing bradyzoites. The cyst can exert pressure on surrounding tissues, potentially triggering local inflammation. This process can also induce delayed hypersensitivity reactions, causing tissue damage such as granuloma formation [35,36]. The CST1 protein in the cyst wall, a glycoprotein, plays a crucial role in stabilizing the cyst wall and shielding it from host immune responses [37,38]. Due to its ease of expression, purification, and good serum reactivity, the CST1 antigen is a representative marker for detecting chronic *T. gondii* infection in blood serum.

To ensure the representativeness of this study population, we performed random sampling within the AD patient group at the Shanghai Mental Health Center. As a prominent mental health medical institution in China, the center’s patient demographics mainly consist of individuals from Shanghai and surrounding areas, indicating a certain geographical limitation. Nonetheless, this can still reflect some characteristics and needs of Chinese patients with mental disorders. Age is a crucial factor related to AD. Therefore, to emphasize the correlation between *T. gondii* infection and AD, we controlled the age of the control group to be above 55. However, it should be noted that this approach also weakened the actual effect of age on the development of AD, because by controlling for age, the age effect in the model is no longer considered the main factor influencing AD onset, while the contribution of CST1 is relatively enhanced. As a result, the impact of age on AD development is somewhat underestimated or masked. Additionally, to better explore the impact of *T. gondii* infection on AD, we selected an appropriate statistical model to minimize the influence of other factors. We first compared the effects of including and excluding the CST1 variable in the model (Table 2), and then evaluated the performance of the model after incorporating various interaction terms, which allowed us to determine the final model (Table 3). Regarding the inclusion of the sex variable in the final model, despite not being statistically significant in the univariate logistic regression analysis, we considered the fact that, in China, data show that among Alzheimer’s and other dementia patients females have higher incidence (1188.9/100,000) and mortality (30.8/100,000) rates compared to males, who have an incidence rate of 669.3/100,000 and a mortality rate of 14.6/100,000 [39]. Therefore, the sex variable was still included in our final prediction model.

The univariate and multivariate logistic regression results indicated that an elevated level of antibodies against the *T. gondii* CST1 antigen is a risk factor for AD (*p* < 0.01). The nomogram suggested that CST1 antibody levels can serve as a predictive marker for AD. The results from the ROC curves demonstrated that the model has good discriminatory power and can effectively distinguish between individuals with AD and those without. The calibration curve results further demonstrated that the model performed well after incorporating CST1 antibody levels, gender, and age into the final model. The model fitted reasonably well on the validation set and was able to estimate prediction probabilities accurately without significant overfitting. Although the results suggest that the model exhibits good predictive ability to some extent, and that CST1 antibody levels can enhance the predictive accuracy of the model, it should be noted that external validation is lacking, as the calibration curve was generated using bootstrap internal validation. Additionally, from a statistical perspective, the sample size of 233 (109 Alzheimer’s patients and 114 healthy controls) is generally sufficient to support basic multivariate analysis and model development in an exploratory study. For standard logistic regression models or other fundamental statistical analyses, this sample size provides a reasonable level of statistical power. However, given the complexity of this study, the large number of Alzheimer’s patients in China, and the number of variables involved (such as gender, age, and CST1 levels), the sample size may still be insufficient to accurately capture all the potential interaction effects and subtle differences. Furthermore, issues related to sample representativeness, especially within the Alzheimer’s patient group, may impact the generalizability of the results. Therefore, while the current sample size is adequate for preliminary analysis, future research should aim to increase the sample size and conduct external validation to ensure the robustness and generalizability of the findings. Above all, these findings should be interpreted with caution, and further studies with larger, externally validated samples are needed to confirm the generalizability and robustness of the model. Further incorporation of more relevant variables, such as genetic history, lifestyle, and other biomarkers, is also needed. Overall, we believe our study remains exploratory, highlighting the potential impact of CST1 antibody levels on Alzheimer’s disease development.

In conclusion, this study confirms that *Toxoplasma gondii* infection is a risk factor for the development of AD, further validating the correlation between *Toxoplasma gondii* infection and AD, consistent with previous research [6,40,41,42]. This study validates the serum antibody level of the *T. gondii* CST1 antigen as an indicator of chronic *Toxoplasma* infection, which may have significant implications for predicting AD onset. However, limitations, such as the small sample size and the regional representativeness of the population sampled, suggest a need for further, large-scale, multicenter studies.

## Figures and Tables

**Table 1:** Baseline demographic characteristics of this study population.

	Total Subjects (*n* = 223)		Test Statistic Value	*p* Value
	Alzheimer (*n* = 109)	Healthy (*n* = 114)		
Age, years	76.9 ± 7.3	70.7 ± 7.2	W = 3454	<0.001
Female sex	68 (62.4)	54 (47.4)	X-squared = 4.483	0.034

Values are mean ± SD or n (%).

**Table 2:** Comparison between the models with and without CST1.

Evaluation Metric	Model with CST1	Model Without CST1
Sensitivity	0.9032	0.7097
Specificity	0.8286	0.6571
False Positive Rate	0.1714	0.3429
False Negative Rate	0.0968	0.2903
Accuracy	0.8636	0.6818
Balanced Accuracy	0.8659	0.6834
Kappa	0.7278	0.3648
Positive Predictive Value (PPV)	0.8235	0.6471
Negative Predictive Value (NPV)	0.9062	0.7188

**Table 3:** Results for multiple multivariate logistic regression models.

Model	Variable	OR	*p*-Value
Model1	intercept	0.00000408	<0.001
	age	1.12	<0.001
	sex (female)	1.15	0.738
	CST1	4.75	<0.001
Model2	intercept	0.00000376	<0.001
	age	1.12	<0.001
	sex (female)	1.36	0.785
	CST1	4.98	<0.001
	sex*CST1	0.926	0.874
Model3	intercept	0.0555	0.637
	age	0.987	0.877
	sex (female)	0.717	0.778
	CST1	0.0567	0.301
	sex*CST1	1.28	0.642
	age*CST1	1.06	0.110
Model4	intercept	0.00508	0.501
	age	1.02	0.847
	sex (female)	6.74	0.880
	CST1	1.35	0.932
	sex*CST1	0.00566	0.346
	age*CST1	1.02	0.723
	age*sex	0.961	0.822
	age*sex*CST1	1.08	0.314

## Data Availability

The original contributions of this study are included in the article/Supplementary Materials section. The data and calculations supporting the results of the article can be provided in supplementary documents, where applicable, with hyperlinks to the publicly archived datasets analyzed or generated during the research period.

## Supplementary Materials

The following supporting information can be downloaded at: https://www.mdpi.com/article/10.3390/pathogens13111021/s1.
