# Supplementary material for: Analysis of the Correlation Between Toxoplasma gondii Seropositivity and Alzheimer’s Disease
Source: Pathogens. 2024 Nov 20;13(11):1021. doi: 10.3390/pathogens13111021 (PMC11597115; doi:10.3390/pathogens13111021)
Supplement: Supplementary file 1 [file pathogens-13-01021-s001.zip › pathogens-3276990-supplementary/supplementary material/Supplementary file 1.pdf]

Ⅰ：Statistical analysis of demographic characteristics

```
# Load the necessary libraries
library(dplyr)
# Calculate the mean and standard deviation of age in the control and case groups
age_stats <- data %>%
  group_by(status) %>%
  summarise(
    age_mean = mean(age, na.rm = TRUE),
    age_sd = sd(age, na.rm = TRUE)
  )
# Calculation of the number and percentage of females in the group
sex_stats <- data %>%
  group_by(status) %>%
  summarise(
    female_count = sum(sex == "female", na.rm = TRUE),
    female_percentage = mean(sex == "female", na.rm = TRUE) * 100
  )
# Combining age statistics and gender statistics
summary_stats <- left_join(age_stats, sex_stats, by = "status")
print(summary_stats)

# Statistical tests
# 1. Test for difference in age between groups
# normality test
shapiro.test(data$age[data$status == 0])  # Normality test for the normal group
shapiro.test(data$age[data$status == 1])  # Normality test for the AD group

# The AD group did not fit the normal distribution, using the Mann-Whitney U test
wilcox_test_result <- wilcox.test(age ~ status, data = data)
print(wilcox_test_result)

# 2. Test for gender differences between groups
# Use the chi-squared test (Chi-squared test) to test whether there is a significant difference in gender distributions
chi_sq_test_result <- chisq.test(table(data$status, data$sex))
print(chi_sq_test_result)
```

Ⅱ：Categorical histogram

```r
# Load the necessary libraries
library(readxl)
library(ggplot2)
library(dplyr)

# retrieve data
data <- read_excel("/mnt/data/data.xlsx")

# Ensure that the status column is factored to facilitate group mapping
data$status <- factor(data$status, levels = c(0, 1), labels = c("Control", "AD"))
# Setting the number of intervals
bins <- 40
# Plot the histogram of CST1
cst1_plot <- ggplot(data, aes(x = CST1, fill = status, group = status)) +
  geom_histogram(bins = cst1_bins, position = "identity", alpha = 0.6) +
  labs(title = "CST1 Distribution by Group", x = "CST1", y = "Count") +
  theme_minimal() +
  theme(
    plot.title = element_text(hjust = 0.5, size = 14, face = "bold"),
    axis.text = element_text(size = 12),
    axis.title = element_text(size = 13, face = "bold"),
    legend.title = element_blank(),
    legend.text = element_text(size = 12)
  ) +
  scale_fill_manual(values = c("Control" = "#FFD700", "AD" = "#4169E1")) # yellow and blue

# Plotting the Histogram of CrudeAntigen
crudeantigen_plot <- ggplot(data, aes(x = CrudeAntigen, fill = status, group = status)) +
  geom_histogram(bins = crudeantigen_bins, position = "identity", alpha = 0.6) +
  labs(title = "CrudeAntigen Distribution by Group", x = "CrudeAntigen", y = "Count") +
  theme_minimal() +
  theme(
    plot.title = element_text(hjust = 0.5, size = 14, face = "bold"),
    axis.text = element_text(size = 12),
    axis.title = element_text(size = 13, face = "bold"),
    legend.title = element_blank(),
    legend.text = element_text(size = 12)
  ) +
  scale_fill_manual(values = c("Control" = "#FFD700", "AD" = "#4169E1")) # yellow and blue
```

# Display Graphics

```
print(cst1_plot)
print(crudeantigen_plot)
```

Ⅲ：Code for data cleaning, building logistic regression models, goodness-of-fit tests, plotting ROC curves and calibration curves, implemented in R version 4.4.1

1. Setting up the working environment

```
rm(list=ls())
setwd("D:/sup1")
```

2. Data loading and cleansing

```
library(readxl)
data1 <- read_excel("C:/Users/Jacob Wang/Desktop/ELASA /data1.xlsx",
col_types = c("numeric", "text", "numeric","text", "numeric",
"numeric"))
View(data1)
```

3. install package “tidyverse”

```
library(tidyverse)
colnames(data1)
```

4. Factorization of the gender variable

```
data1$sex<-factor(data1$sex,labels=c("female","male"))
str(data1)
```

5. Setting seeds to ensure reproducibility of data analysis

```
set.seed(0119)
```

6. The dataset was divided into a training set and a validation set in a ratio of 7:3 to clarify whether overfitting had occurred and to prevent erroneous conclusions from being drawn. "replace=F" represented no-putback sampling

```
train<-sample(data1$id,floor(nrow(data1)*0.7),replace = F)
data_train <- filter(data1,data1$id %in% train)
data_test <- filter(data1,!data1$id %in% train)
```

7. Variable screening and modeling

7.1 univariate logistic regression

```
monofactor<-list()
for(i in c(3:6)){
print(i)
f<-glm(as.formula(paste0("status~",colnames(data1)[i])),
data=data_train)
monofactor[[i-2]]<-summary(f)
}
monofactor
```

7.2 multivariate logistic regression

```
multi<-glm(status~sex + age + CST1,
data=data_train,
family=binomial())
summary(multi)

library(rms)
```

8. Packaging data

```
dd<-datadist(data_train)
options(datadist='dd')
```

9. Building the final logistic model

```
final<-lrm(status~sex + age + CST1,
data=data_train,x=T,y=T)
final
```

10. Hmisc package loading, validation of final model using validate function

```
library(Hmisc)
v<-validate(final,
method="boot",
B=1000,
Dxy=T)
```

11. The Dxy index values were extracted from the validation results and the corrected C-index (a measure of prediction accuracy) was calculated

```
Dxy=v[rownames(v)=="Dxy",colnames(v)=="index.corrected"]
bias_correct_c_index<-abs(Dxy)/2+0.5
bias_correct_c_index
```

12. Hosmer-Lemeshow goodness-of-fit test

```
library(ResourceSelection)
hl<-glm(status~sex + age + CST1,
data=data_train,
family=binomial())
hoslem.test(data_train$status,
fitted(hl),
g=10)
```

13. Mapping Nomograms

```
nom1<-nomogram(final,
fun=plogis,
lp=F,
fun.at=c(0.01,0.05,0.5,0.9,0.99),
funlabel="Mortality risk")
plot(nom1)
```

14. Plotting the ROC curve for the training set

```
library(pROC)
```

```
roc_train<-roc(status~
predict(final),
data=data_train)
plot(roc_train,
print.auc=TRUE,
auc.polygon=TRUE,
max.auc.polygon=TRUE,
auc.polygon.col="skyblue",
main="ROC Curve(Training set)",
col="red",
identity.col="blue",
identity.lty=1,
identity.lwd=1)
roc_train$auc
ci(roc_train)
cutoff_train<-coords(roc_train,"best",ret="threshold")
cutoff_train
spe_train<-coords(roc_train,"best")$specificity
spe_train
sen_train<-coords(roc_train,"best")$sensitivity
sen_train
```

15. Plotting the ROC curve for the Validation set

```
final_test<-lrm(status~sex + age + CST1,
data=data_test,x=T,y=T)
final_test
roc_test<-roc(status~
predict(final_test),
data=data_test)

plot(roc_test,
print.auc=TRUE,
auc.polygon=TRUE,
max.auc.polygon=TRUE,
auc.polygon.col="skyblue",
main="ROC Curve(Validation set)",
col="red",
identity.col="blue",
identity.lty=1,
identity.lwd=1)
roc_test$auc
ci(roc_test)
```

16. Plotting calibration curves for the training set

```
cal_train<-calibrate(final,
method='boot',
```

```
m=50,
B=1000)
plot(cal_train,
Xlim=c(0,1),
xlab="Predicted Probability",
ylab="Observed Probability",
legend=FALSE,
subtitles=FALSE)
abline(0,1,col="black", lty=2, lwd=2)
abline(0,1,col="black", lty=1, lwd=1)
abline(0,1,col="black", lty=2, lwd=2)
lines(cal_train[,c("predy","calibrated.orig")],
type ="l",lwd=2,col="red",pch=16)
lines(cal_train[,c("predy","calibrated.corrected")],
type ="l",lwd=2,col="green",pch=16)
legend(0.55,0.25,
c("Ideal","Apparent","Bias-corrected"),
lty = c(2,1,1),
lwd = c(2,1,1),
col = c("black","red","green"),
bty = "n")
```

17. Plotting calibration curves for the validation set

```
cal_test<-calibrate(final_test,
method='boot',
m=50,
B=1000)
plot(cal_test,
Xlim=c(0,1),
xlab="Predicted Probability",
ylab="Observed Probability",
legend=FALSE,
subtitles=FALSE)
abline(0,1,col="black", lty=2, lwd=2)
lines(cal_test[,c("predy","calibrated.orig")],
type ="l",lwd=2,col="red",pch=16)
lines(cal_test[,c("predy","calibrated.corrected")],
type ="l",lwd=2,col="green",pch=16)
legend(0.55,0.25,
c("Ideal","Apparent","Bias-corrected"),
lty = c(2,1,1),
lwd = c(2,1,1),
col = c("black","red","green"),
bty = "n")
```

Ⅳ：Sensitivity, specificity, false-positive rate, and false-negative rate were calculated for the model with and without CST1 respectively

```
# Load the necessary libraries
library(readxl)
library(caret)
library(pROC)
library(rms)
# Set the number of seeds to ensure that the results are reproducible
set.seed(0119)
# Stratified sampling to ensure rational allocation of control and AD sets for training and test sets
trainIndex <- createDataPartition(data$status, p = 0.7, list = FALSE)
trainData <- data[trainIndex, ]
testData <- data[-trainIndex, ]
#model with CST1
model_with_CST1 <- glm(status ~ age + sex + CST1, data = trainData, family = binomial)
#model without CST1
model_without_CST1 <- glm(status ~ age + sex, data = trainData, family = binomial)
# Predictions on the validation set (containing the CST1 model)
pred_probs_with_CST1 <- predict(model_with_CST1, newdata = testData, type = "response")
pred_labels_with_CST1 <- ifelse(pred_probs_with_CST1 > 0.5, 1, 0)
# Predictions on the test set
predictions_with_CST1 <- predict(model_with_CST1, testData, type = "response")
predictions_without_CST1 <- predict(model_without_CST1, testData, type = "response")
# Convert predictions to binary classification
predicted_classes_with_CST1 <- ifelse(predictions_with_CST1 > 0.5, 1, 0)
predicted_classes_without_CST1 <- ifelse(predictions_without_CST1 > 0.5, 1, 0)
# Calculate sensitivity, specificity, false positive rate, false negative rate
conf_matrix_with_CST1 <- confusionMatrix(factor(predicted_classes_with_CST1), factor(testData$status))
conf_matrix_without_CST1 <- confusionMatrix(factor(predicted_classes_without_CST1), factor(testData$status))

#print results
cat("model with CST1：\n")
print(conf_matrix_with_CST1)
cat("\n model without CST1：\n")
print(conf_matrix_without_CST1)
```

Ⅴ：Multiple multivariate models considering interactions

```
# Load the necessary packages
library(dplyr)
library(broom)
# Suppose the data frame is 'data' and contains 'status', 'age', 'sex', 'CST1'
# Define a function to construct and output model results
run_models <- function(data, CST1_label) {
  cat("\nResults for", CST1_label, "\n")
  # 1. Modeling 1: including age, sex and CST1
  model1 <- glm(status ~ age + sex + CST1, data = data, family = binomial)
  # 2. Modeling 2: Including an interaction term between sex and CST1
  model2 <- glm(status ~ age + sex + CST1 + sex * CST1, data = data, family = binomial)
  # 3. Modeling 3: Adding an interaction term between age and CST1
  model3 <- glm(status ~ age + sex + CST1 + sex * CST1 + age * CST1, data = data, family = binomial)
  # 4. Modeling IV: Adding a Triple Interaction Term for Age, Sex, and CST1
  model4 <- glm(status ~ age + sex + CST1 + sex * CST1 + age * CST1 + sex * age * CST1, data = data, family = binomial)
  # Extract and calculate OR and p-values
  model1_results <- tidy(model1) %>% mutate(OR = exp(estimate)) %>% select(term, OR, p.value)
  model2_results <- tidy(model2) %>% mutate(OR = exp(estimate)) %>% select(term, OR, p.value)
  model3_results <- tidy(model3) %>% mutate(OR = exp(estimate)) %>% select(term, OR, p.value)
  model4_results <- tidy(model4) %>% mutate(OR = exp(estimate)) %>% select(term, OR, p.value)
  # print results
  cat("Model 1 Results:\n")
  print(model1_results)

  cat("\nModel 2 Results:\n")
  print(model2_results)

  cat("\nModel 3 Results:\n")
  print(model3_results)

  cat("\nModel 4 Results:\n")
  print(model4_results)
}

# Using the original CST1 variable
```

```
run_models(data, "Original CST1")
```
# Variables after multiplying by 10 using CST1
```
data_10 <- data %>% mutate(CST1 = CST1 * 10)
run_models(data_10, "CST1 * 10")
```
# Variables after multiplying by 100 using CST1
```
data_100 <- data %>% mutate(CST1 = CST1 * 100)
run_models(data_100, "CST1 * 100")
```

Ⅵ：Parallel experimental correlation test：

# 1. Import data:
```
library(readxl)
  data <- read_excel("C:/Users/Jacob Wang/Desktop/data.xlsx")
View(data)
str(data)
```
# 2. Plotting scatter plots and adding fitted line
```
plot(data$CST1, data$CrudeAntigen, main = "Scatter Plot with Fit Line", xlab = "CST1", ylab = "Crude Antigen")> abline(lm(CrudeAntigen ~ CST1, data = data), col = "red")
```
# 3. Calculation of regression models
```
model <- lm(CrudeAntigen ~ CST1, data = data)
```
# 4. Calculating statistics and adding them directly to the charts
```
cat("R-squared:", summary(model)$r.squared, "\n")
R-squared: 0.6241556
r_squared <- summary(model)$r.squared
r_value <- sqrt(r_squared)
text(x = min(data$CST1), y = max(data$CrudeAntigen), + labels = paste0("R: ", round(r_value, 3), +    "\nR²: ", round(r_squared, 3)),+ pos = 4, col = "black")
```

Ⅶ：10-fold cross validation
```
library(readxl)> data <- read_excel("C:/Users/Jacob Wang/Desktop/data.xlsx")
> View(data)
> library(MASS)
> library(caret)
> library(pROC)
> library(ggplot2)
> library(dplyr)
> data$sex <- factor(data$sex, labels = c("1", "2"))
> str(data)
set.seed(123)
> ind <- sample(2, nrow(data), replace = TRUE, prob = c(0.7, 0.3))
> train <- data[ind == 1, ]
> test <- data[ind == 2, ]
> train$status <- factor(train$status, levels = c(0, 1), labels = c('No', 'Yes'))
> train.control <- trainControl(
```

```
                                              method = "cv",
                                            number = 10,
                                            classProbs = TRUE,
                                            savePredictions = TRUE,
                                            summaryFunction = twoClassSummary
                                            )
> names(train)


model <- train(
status ~ age + sex + CST1,
data = train,
method = "svmRadial",
trControl = train.control,
metric = "ROC" )
> print(model)

> folddata <- data.frame(model$pred)
> accuracy_results <- folddata %>%
  group_by(Resample) %>%
summarise(
  cm = list(confusionMatrix(pred, obs)),
accuracy = cm[[1]]$overall['Accuracy']
              )
> print(accuracy_results)

roc_results <- list()
for (i in 1:10) {
fold <- subset(folddata, Resample == paste0("Fold", sprintf("%02d", i)))
       roc_results[[i]] <- roc(fold$obs, fold$Yes)
       cat(paste0("Fold ", i, " AUC: ", round(roc_results[[i]]$auc, 3), "\n"))
         }
```

Results:(Setting levels: control = No, case = Yes

```
Setting direction: controls < cases
Fold 1 AUC: 0.552
Setting levels: control = No, case = Yes
Setting direction: controls < cases
Fold 2 AUC: 0.713
Setting levels: control = No, case = Yes
Setting direction: controls < cases
Fold 3 AUC: 0.662
Setting levels: control = No, case = Yes
Setting direction: controls > cases
Fold 4 AUC: 0.463
```

```
Setting levels: control = No, case = Yes
Setting direction: controls < cases
Fold 5 AUC: 0.696
Setting levels: control = No, case = Yes
Setting direction: controls < cases
Fold 6 AUC: 0.628
Setting levels: control = No, case = Yes
Setting direction: controls < cases
Fold 7 AUC: 0.828
Setting levels: control = No, case = Yes
Setting direction: controls < cases
Fold 8 AUC: 0.706
Setting levels: control = No, case = Yes
Setting direction: controls < cases
Fold 9 AUC: 0.689
Setting levels: control = No, case = Yes
Setting direction: controls < cases
Fold 10 AUC: 0.596)

> mean_roc <- roc(folddata$obs, folddata$Yes)
> cat("Mean AUC: ", round(mean_roc$auc, 3), "\n")
    (result:Mean AUC:   0.632)
roc_data <- data.frame(
                    specificity = 1 - roc_results[[1]]$specificities,
                    sensitivity = roc_results[[1]]$sensitivities,
                    Resample = "Fold01"
                     )
> for (i in 2:10) {
roc_data <- rbind(roc_data, data.frame(
specificity = 1 - roc_results[[i]]$specificities,
sensitivity = roc_results[[i]]$sensitivities,
  Resample = paste0("Fold", sprintf("%02d", i))
))
}

roc_data <- rbind(roc_data, data.frame(
    specificity = 1 - mean_roc$specificities,
    sensitivity = mean_roc$sensitivities,
    Resample = "Mean"
    ))
```

# Plot ROC curves for all folds

```
> ggplot(roc_data, aes(x = specificity, y = sensitivity, color = Resample))+
geom_line() +
theme_bw() +
```

```
xlab("1-Specificity") +
ylab("Sensitivity") +
labs(title = "10-Fold Cross-Validation ROC Curves") +
theme(
    plot.title = element_text(hjust = 0.5, size = 15, face = "bold"),
    axis.text = element_text(size = 12, face = "bold"),
    axis.title = element_text(size = 12, face = "bold"),
    legend.title = element_blank(),
    legend.text = element_text(size = 7.5, face = "bold"),
    legend.position.inside = c(0.8, 0.2),
    legend.background = element_blank(),
    panel.border = element_rect(color = "black", linewidth = 1),
    panel.background = element_blank()
    ) +
    annotate(geom = 'segment', x = 0, y = 0, xend = 1, yend = 1, colour = 'grey', linetype = 'dotdash')
```
